# The Role of River Morphodynamic Disturbance and Groundwater Hydrology As Driving Factors of Riparian Landscape Patterns in Mediterranean Rivers

**DOI:** 10.3389/fpls.2017.01612

**Published:** 2017-09-20

**Authors:** Rui Rivaes, António N. Pinheiro, Gregory Egger, Teresa Ferreira

**Affiliations:** ^1^Forest Research Centre, Instituto Superior de Agronomia, Universidade de Lisboa Lisbon, Portugal; ^2^Civil Engineering Research Innovation and Sustainability Centre, Instituto Superior Técnico, Universidade de Lisboa Lisbon, Portugal; ^3^Environmental Consulting Klagenfurt Klagenfurt, Austria; ^4^Institute of Floodplain Ecology, Karlsruhe Institute of Technology Karlsruhe, Germany

**Keywords:** riparian vegetation, riparian drivers, fluvial disturbances, Mediterranean, confirmatory factor analysis

## Abstract

Fluvial disturbances, especially floods and droughts, are the main drivers of the successional patterns of riparian vegetation. Those disturbances control the riparian landscape dynamics through the direct interaction between flow and vegetation. The main aim of this work is to investigate the specific paths by which fluvial disturbances, distributed by its components of groundwater hydrology (grndh) and morphodynamic disturbance (mrphd), drive riparian landscape patterns as characterized by the location (position in the river corridor) and shape (physical form of the patch) of vegetation patches in Mediterranean rivers. Specifically, this work assesses how the different components of fluvial disturbances affect these features in general and particularly in each succession phase of riparian vegetation. grndh and mrphd were defined by time and intensity weighted indexes calculated, respectively, from the mean annual water table elevations and the annual maximum instantaneous discharge shear stresses of the previous decade. The interactions between riparian landscape features and fluvial disturbances were assessed by confirmatory factor analysis using structural equation modeling. Two hypothetical models for patch location and shape were conceptualized and tested against empirical data collected from 220 patches at four different study sites. Both models were successfully fitted, meaning that they adequately depicted the relationships between the variables. Furthermore, the models achieved a good adjustment for the observed data, based on the evaluation of several approximate fit indexes. The patch location model explained approximately 80% of the patch location variability, demonstrating that the location of the riparian patches is primarily driven by grndh, while the mrphd had very little effect on this feature. In a multigroup analysis regarding the succession phases of riparian vegetation, the fitted model explained more than 68% of the variance of the data, confirming the results of the general model. The patch shape model explained nearly 13% of the patch shape variability, in which the disturbances came to have less influence on driving this feature. However, grndh continues to be the primary driver of riparian vegetation between the two disturbance factors, despite the proportional increase of the mrphd effect to approximately a third of the grndh effect.

## Introduction

Riparian ecosystems are dynamic systems found in flood-prone areas along rivers. They represent the transition between the aquatic and terrestrial ecosystems (Naiman and Décamps, 1997) and play a decisive role in riverine integrity (Van Looy et al., 2013). Riparian ecosystems rely greatly on the characteristics of the flow regime (e.g., Poff et al., 1997) and are notably susceptible to flow regime changes (e.g., Bejarano et al., 2012). The natural inter- and intra-annual variability of the flow regime determines the highly variable fluvial disturbances to which riparian vegetation respond structurally in the medium- to long-term (Whited et al., 2007). Therefore, fluvial disturbances, i.e., the disruption imposed by the seasonal sequence of river flooding and drying (particularly their intensity and spatial extent), are the main drivers of the ecological succession of riparian vegetation (Corenblit et al., 2007). Accordingly, fluvial disturbances control the creation, development and recycling of vegetation patches (Bendix and Hupp, 2000). Fluvial disturbances are also important to maintain the ecological quality of riparian woodlands by providing services like reducing the occurrence of exotic plant species (Greet et al., 2015), promoting species diversity and richness in riparian bird communities (Merritt and Bateman, 2012) or even controlling the understory vegetation (Kamisako et al., 2007). Moreover, the dynamics of the disturbance pattern (Formann et al., 2013) substantiates the river processes that directly impact the riparian vegetation in its interactions with the surface and groundwater river flow (Camporeale and Ridolfi, 2006). The river stage is a proxy for fluvial disturbances. It fluctuates in the form of flood pulses (Junk et al., 1989) according to the hydraulic and hydrologic characteristics of the river. This effects the succession dynamics of riparian vegetation both physically and physiologically (Blom and Voesenek, 1996; Poff et al., 1997; Kozlowski, 2002; Džubáková et al., 2015) due to flood-induced stress through vegetation entrainment, uprooting, burial or anoxia (e.g., Friedman and Auble, 1999; Bendix and Hupp, 2000; Edmaier et al., 2011; Bendix and Stella, 2013). Also a consequence of the river stage is the oscillation of the groundwater level (Jansson et al., 2007). This determines a physiological effect by water stress control on plant growth and survival, affecting species differently according to their greater or lesser dependency on the connection of the root system with the groundwater table (Stromberg et al., 1996; Shafroth et al., 2000; Cooper et al., 2003; Baird et al., 2005; Lowry et al., 2011). The combination of all of these morphodynamic and physiological factors determines a multiplicity of physical habitats that control the presence of riparian flow response guilds (Merritt et al., 2010; Sarr et al., 2011; Bejarano et al., 2012) in which discrete units of homogeneous vegetation occur in different succession phases. These succession phases are characterized by stands of specific ages, structural features and species compositions (Stanford et al., 2005). At a local scale, those are expected to be affected mainly by stream power and depth to groundwater (Bendix, 1999; Cooper et al., 1999; Bendix and Stella, 2013). Consequently, the riparian succession phase is a reliable indicator of the underlying hydraulic processes of fluvial disturbances, in which floods and droughts are the major stressors (Poff et al., 1997; Lytle and Poff, 2004; Stromberg and Boudell, 2013).

Currently, the function of riparian ecosystems and their interactions with their driving forces is well-understood (e.g., Gregory et al., 1991; Nilsson and Svedmark, 2002; Scott et al., 2005; Corenblit et al., 2007). However, the specific paths by which the drivers affect the riparian landscape have scarcely been investigated, especially regarding the local disturbances at a reach scale, or for Mediterranean flow regimes. In this context, this study aimed to investigate the effect of fluvial disturbances on two central elements of landscape ecology, the location (position in the river corridor) and shape (physical form of the patch) of riparian vegetation patches. Indeed, the patch location is important for the spatial characterization authenticity in patch-occupancy models (Fahrig, 2007) while the patch shape indicates the effect on many important ecological processes, such as colonization and growth (Hardt and Forman, 1989), landscape connectivity (Buechner, 1989), and most of all, ecosystem integrity associated with edge effects (e.g., Imre and Bogaert, 2004). As specific objectives, we were particularly interested in addressing the following questions. Can fluvial disturbances, particularly its components of mrphd and grndh, affect the location and shape of riparian patches? How do these different components of fluvial disturbances affect these features? Is this effect on riparian vegetation similar in every succession phase? In order to address these questions we started by performing a thorough literature review to support the specification of our theoretical model constructs. After model specification, field surveys were carried out in three Mediterranean rivers to collect the necessary vegetation data. Finally, following data treatment, we analyzed the paths by which fluvial disturbances drive riparian vegetation patterns. By these means, we attempted to provide essential knowledge on flow regime management for an enhanced riparian restoration, which is an indispensable and most promising way to restore natural processes in degraded rivers (Palmer et al., 2014).

## Materials and Methods

The interactions between riparian spatial patterns and fluvial disturbances were analyzed by confirmatory factor analysis with SEM. SEM is a multivariate statistical modeling technique that combines factor analysis and regression analysis to validate fundamental theories with empirical data, and therefore provides a deeper analysis than traditional statistical methodologies (Malaeb et al., 2000). Furthermore, SEM has some interesting characteristics that overcome the standard first generation of multivariate statistical techniques, which are very useful for this kind of research. To begin with, SEM enables the incorporation of latent variables (also known as factors) in the analysis and tests the theoretical model constructs that they represent. Latent variables represent theoretical concepts that cannot be directly measured, such as ecosystem health or habitat suitability, but which are manifested by directly measurable variables (indicators or observed variables) that show the underlying variability of these theoretical concepts (Beaujean, 2014). SEM also enables the possibility of the simultaneous investigation of all of the effects and responses of the variables in the model construct, therefore providing a comprehensive picture of the system as a whole instead of the processes that comprise it. Finally, SEM takes measurement errors into account and hence offers better consistency and precision in parameter estimation (McCoach et al., 2007).

### Model Specification

The specification of the model consists of transforming the researcher’s perceptions into the formal configuration of a structural equation model. This transformation is the most important and complex task in SEM because each of the following steps is grounded on the premise that the model designed is properly specified and that only a correctly specified model can properly test the researcher’s hypotheses (Ntoumanis and Myers, 2016).

**FIGURE 1:**
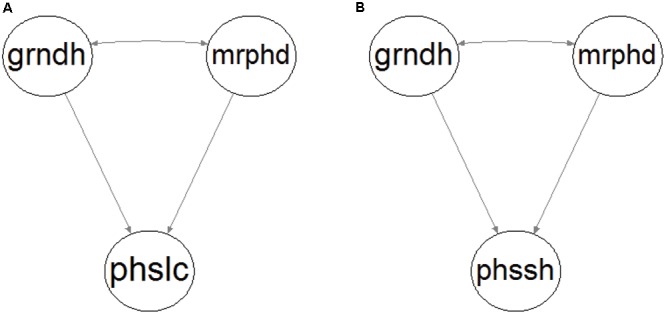
Conceptual model construct of riparian patch location **(A)** and shape **(B)**. Ellipses represent the following latent variables: groundwater hydrology (grndh), morphodynamic disturbance (mrphd), patch location (phslc), and patch shape (phssh). Single-headed arrows stand for direct relationships and double-headed arrows between variables for existing unexplained correlations.

According to the literature review presented in the Introduction, fluvial disturbance directly impacts riparian vegetation in two major ways, mrphd and physiological stress. A mrphd means surface flow-derived processes that cause physical vegetation damage, sediment burial or uprooting, and entrainment (e.g., Friedman and Auble, 1999; Edmaier et al., 2011; Bendix and Stella, 2013). Physiological stress is a consequence of the grndh and impacts riparian vegetation as water stress caused by lowering of the level of groundwater table during a drought or by anoxia during flood periods (e.g., Stromberg et al., 1996; Shafroth et al., 2000; Cooper et al., 2003; Baird et al., 2005; Lowry et al., 2011). The current patch mosaic (i.e., the location and shape of the patches) of riparian vegetation is therefore the outcome of the existing conditions in the habitat, mainly as a consequence of the historical flow regime (Pettit et al., 2001). Consequently, we based ourselves on the literature review in the Introduction and our expert knowledge of riparian ecosystems to conceptualize two theoretical model constructs to address these research questions (**Figure 1**).

Morphodynamic disturbance and groundwater hydrology are the exogenous variables that depict the flow regime and have a direct effect on the endogenous variables patch location (phslc; **Figure 1A**) and patch shape (phssh; **Figure 1B**). These variables cannot be measured directly, so they are considered latent variables expressed by manifest variables that are measurable. Because both mrphd and grndh are controlled by the flow regime, they are expected to be correlated to some extent. The physiological effects of the duration of flooding on vegetation were not expressly accounted for by the models because they were not expected to play an important role in the riparian ecological succession in the Mediterranean watersheds considered in this study. The characteristic flashiness of the pluvial flow regime defines very short flood durations in small catchment areas, and furthermore, are restricted to the winter (Tockner et al., 2009), which is the dormant vegetation season, when floods have less of an effect (Crawford, 2003).

Finally, the model constructs were hypothesized to be parsimonious as possible, and the latent variable indicators were reduced by the maximum amount. In fact, single or only a small number of latent variable indicators are normal in the natural sciences due to the nature of the data (Grace, 2006). This strategy was adopted not only to avoid identification problems (Schumacker and Lomax, 2010) but also to make use of only one or two of the best indicators as recommended and often sufficient (Hayduk and Littvay, 2012), as well as for the sake of the simplicity that must be sought for any ecological model (Jackson et al., 2000).

### Model Identification

The objective of model identification is to determine if a theoretical model construct enables the unique estimation of the requisite parameters from existing non-redundant information in the data. Consequently, to assure the identification of the proposed models, the factor loadings of the latent variables with only one indicator were set to 1 and the corresponding indicator error variance was set to 0 (Beaujean, 2014). Hence, 4 factor loadings exist for the specified models, 4 measurement error variances, 2 path coefficients, 1 correlation between the latent variables and 3 equation error variances – a total of 14 parameters that must be estimated. Each of the model constructs has only 8 free parameters (relationship coefficients to be estimated from the collected data) and 4 observed variables, implying that there are (8 × 4)/2 = 16 pieces of non-redundant information. Because only 14 parameters must be estimated, the models are considered overidentified and therefore the model identification is verified. Additionally, to have sufficient variability to estimate the model parameters, the sample size (N) should follow the rule of at least 20 cases for each free parameter that must be estimated (Jackson, 2003). Accordingly, in this situation N should be at least 160 observations. Notwithstanding, the usual minimum sample size for SEM studies is approximately 200 cases (Kline, 2011).

### Data collection

**FIGURE 2:**
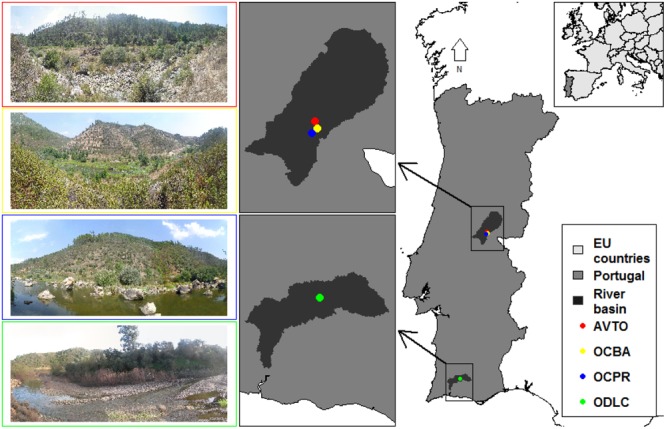
Location and characterization of the study sites AVTO (red), OCBA (yellow), OCPR (blue) and ODLC (green).

Four study sites were selected in natural conditions of riparian vegetation and flow regime (**Figure 2**). Upstream of the study sites, the main land uses in the watersheds are planted forests and natural shrublands, with very sparse villages and no noteworthy industry. In all cases, the flow regime was considered unregulated and typically Mediterranean, with a low winter flow interspersed by flash floods, and a very low and often intermittent summer flow (Bonada and Resh, 2013). The woody riparian species composition was similar in the study sites, comprising mainly willows (*Salix* spp.) and ashes (*Fraxinus angustifolia*). Notwithstanding, the four sites encompassed diverse fluvial geomorphologies, watershed features and river sizes (**Table 1**).

**Table 1:** Characterization of the study sites.

		OCBA				OCPR				AVTO				ODLC			
Watershed area (km^2^)		779				1037				177				186			
Distance do source (km)		63				66				30				34			
Mean discharge (m^3^/s)		7.9				11.0				2.3				3.2			
Mean annual maximum instantaneous discharge (m^3^/s)		322				457				95				116			
Mean slope (m/m)		0.003				0.006				0.012				0.002			
Main substrate type		Boulders				Large boulders				Boulders				Cobbles			
Total succession phase area (%)	IP	37.2				30.5				26.37				25.8			
	PP	23.2				8.0				6.67				5.7			
	ES	11.3				22.1				16.2				18.6			
	EF	28.2				39.4				50.8				49.9			
				
**Species inventory (mean coverage %)^∗^**		**IP**	**PP**	**ES**	**EF**	**IP**	**PP**	**ES**	**EF**	**IP**	**PP**	**ES**	**EF**	**IP**	**PP**	**ES**	**EF**
				
*Alnus glutinosa*		–	0.1	1.8	–	–	0.1	–	–	–	2.1	10.4	–	–	–	–	–
*Cistus* spp.		–	–	–	–	–	–	–	1.2	–	–	–	1.2	0.1	–	–	0.2
*Crataegus monogyna*		–	–	–	7.6	0.3	–	–	0.2	1.3	1.4	1.1	2.0	–	–	0.3	2.3
*Erica* spp.		–	2.4	4.0	8.8	0.3	–	–	16.6	0.1	–	1.4	14.0	–	–	–	–
*Ficus carica*		–	–	–	–	–	–	–	–	–	–	–	–	–	–	–	1.2
*Flueggea tinctoria*		0.5	10.6	21.5	1.9	1.8	4.6	21.1	–	0.3	28.1	26.8	–	–	–	–	–
*Fraxinus angustifolia*		–	0.2	5.1	27.4	0.3	–	0.5	24.6	0.3		3.2	1.6	1.4	4.0	9.0	45.8
*Olea europaea*		–	–	–	–	–	–	–	–	–	–	–	1.0	0.2	–	0.1	10.6
*Phillyrea* spp.		–	–	–	2.0	0.5	–	–	1.2	–	–	–	5.2	–	–	–	0.2
*Pinus pinaster*		–	–	–	0.1	–	–	–	0.4	–	–	–	1.2	–	–	–	–
*Quercus rotundifolia*		–	–	–	–	–	–	–	0.2	–	–	–	1.0	–	–	–	3.5
*Quercus suber*		–	–	–	–	–	–	–	–	–	–	–	–	–	–	–	3.0
*Rosa canina*		–	0.6	1.0	0.8	–	–	–	1.0	1.0	–	0.4	1.0	–	0.1	0.8	1.6
*Salix* spp.		0.5	10.0	41.3	–	1.8	18.0	47.9	–	–	19.6	35.0	0.4	1.4	0.1	27.7	–
*Tamarix africana*		–	–	–	–	–	–	–	–	–	–	–	–	2.9	10.6	22.8	14.5

*^∗^Species inventory only include phanerophyte species with at least 1% coverage in one of the succession phases of the four study sites. All the species mentioned are native.*

A complete survey of the topography and riparian vegetation was performed at each study site. The surveys were done on river reaches 300–500 m long (depending on the river width maintaining a ratio between length and main channel width of 10–20) and extended laterally to the area flooded by a 100-year recurrence interval (normally determining study site widths of approximately 70–110 m). This area was determined by modeling such a flood in each study site using the hydrodynamic model River2D. The topography was surveyed with an elevation detail of 20 cm using both total stations (Leica TPS400) and Global Navigation Satellite Systems (Leica 500 GPS, composed of two double-frequency-to-real-time SR 530 RTK antennas L1 and L2 AT 502). Riparian vegetation surveys were performed using a sub-meter handheld GPS (Ashtech, Mobile Mapper 100) to outline and georeference all the existing vegetation patches. Vegetation inventories in each patch sought to characterize succession phases by vegetation attributes and included phanerophyte species identification (**Table 1**). Then, each vegetation patch was classified by its succession phase, based on the vegetation type and the patch age. The succession phase classification methodology followed Rivaes et al. (2013) in which vegetation types were defined by indicator species and patch age by dendrochronological methods. A total of 220 vegetation patches in the aquatic, bank and floodplain zones were assessed. Four succession phases were found at all the study sites, namely, initial phase (IP), pioneer phase (PP), early successional woodland phase (ES), and established forest phase (EF). Altogether, those succession phases account for the existing ecological succession phases of riparian woodlands in those Mediterranean rivers (García-Arias et al., 2013). In detail, IP is characterized by areas with less than 50% vegetation cover and the absence of woody species. PP is typified by the recruitment areas of woody species and ES is characterized by the presence of well-established softwood pioneer individuals such as willows. EF is found in patches presenting hardwood species such as ashes, along with a well-defined understory stratum (**Table 1**).

The hydraulic parameters were obtained with the River2D model (Steffler et al., 2002) and were used to create the shear stress maps of the annual maximum instantaneous discharges and the mean annual water table elevations. The River2D is a hydrodynamic 2D model based on the depth averaged Saint Venant equations that computes the depth and the discharge intensities in the x-y directions. This tool was developed for application in natural rivers and features wet-dry area solution capabilities by combining surface flow and groundwater flow equations to compute the free elevation above and below the ground. River2D also incorporates a bed resistance model and a transverse shear model. In the former model, bed shear stresses are assumed to be related through the effective roughness height to the magnitude and direction of the depth-averaged velocity. The advantage of using the roughness height as the resistance parameter is that it remains constant over a wide range of depth. In the transverse shear model, the depth-averaged transverse turbulent shear stresses are modeled using a Boussinesq type eddy viscosity formulation. A complete description of the model is provided by Ghanem et al. (1996). Shear stress has been widely used as a fundamental proxy for soil erodibility, mrphd and drag imposed on vegetation by river flows. In natural channels, shear stress is considered to be balanced by three resistance components, namely, viscous drag on the ground surface on particles, pressure drag associated with large non-vegetal boundary roughness and drag on vegetal elements (Temple et al., 1987). The maps produced by River2D had a precision of a quarter of a square meter and were used to compute the MDi and the GWDi developed by Egger et al. (2014, 2016). Both are time and intensity weighted indexes (TIWI) that characterize the processes of grndh and mrphd of the historical flow regime in the past decade at each study site. These indexes are proxies for the long-term ecosystem processes of physiological stress and geomorphic-mechanical disturbance that effect riparian communities and provide a parameter that allows a dynamic analysis of riparian ecosystem patterns. Mean values of MDi and GWDi were calculated for the area covered by each of the vegetation patches recorded. The patch features were obtained using the *ArcGis 9.2* (Environmental Systems Research Institute, 2010) and its *Patch Analyst* extension (Rempel et al., 2012). The relative positioning of the patches in the study sites were characterized by height (THAH) and distance (THAD) to thalweg. The patch shape metrics selected were the patch perimeter (PERIMTR) and the mean patch fractal dimension (MPFD). PERIMTR is an edge metric that provides a measurement of the dimensions and amount of edge created by each patch. This variable significantly affects many ecological phenomena and the analysis of spatial patterns in landscape ecological research (McGarigal and Marks, 1994). The MPFD is a shape metric that represents the geometric complexity of the patches and provides information regarding the formation and quality of the patches (Imre and Bogaert, 2004). Finally, the patch data were compiled and a data matrix was built and uploaded in the R environment (R Development Core Team, 2011) for subsequent data validation and treatment.

### Data Validation and Treatment

The variables were log transformed to remove bias and to bypass model under-identifiability imposed by disproportionate scales (McDonald and Ho, 2002). Outliers for which no justification was found were removed. Data were subsequently checked for compliance for the statistical assumptions for the SEM. Data normality was assessed with the Shapiro–Wilk normality test (Shapiro and Wilk, 1965), supported by an assessment of data sk and ku. Sk and ku were also used to assess the data normality to some extent, providing information about the distribution of the variables, i.e., the symmetry and the “peakedness” of the distribution. In this particular case, even after the data were transformed, some of the variables considered continued to have normality issues and did not pass the Shapiro–Wilk normality test. However, the magnitude of the sk and ku of the variables were always below 0.88 and 1.01, respectively. No consensus exists as yet about the maximum magnitude of sk and ku that does not undermine the reliability of the conclusions about the model fit and the parameter estimates (Finney and DiStefano, 2006) but the most conservative thresholds found in the literature are maximum absolute values of 1 and 1.5, respectively (e.g., Schumacker and Lomax, 2010; Kline, 2011). Furthermore, for large sample sizes (*N* ≥ 40) non-normality is not considered problematic (Pallant, 2002) and can be ignored (Altman and Bland, 1995). Consequently, the data were considered to be normal. The linearity between the variables was assessed using the Harvey-Collier and Rainbow tests for linearity, as well as Ramsey’s RESET test for functional form. The majority of the variable relationships passed all three tests and only two relationships were linear in at least one of the tests with a 99% confidence level. Consequently, the variables were considered to be linearly related. Independence was tested using Moran’s autocorrelation coefficient (Moran, 1950) and the Mantel test (Mantel, 1967) for spatial correlation. The data were considered independent, because both Moran’s coefficient and the Mantel test did not demonstrate any spatial correlation of the riparian succession phases between sites or at each site, with a confidence level of 99%. Homoscedasticity was assessed by a plot analysis of the residuals versus the predicted values. No substantial problems were found by a visual assessment of the plots and therefore, the data was considered homoscedastic. Multicollinearity was evaluated by calculating the variance inflation factor (VIF) for each variable. The VIF is a widely used parameter to determine the degree of multicollinearity between sets of observations (Liao and Valliant, 2012; García et al., 2015; Salmerón Gómez et al., 2016), and provides an indication of the effects of multicollinearity on the variance of the regression coefficients. A threshold of 10 is the most common value used as an indication of multicollinearity and a lower VIF is indicative of inconsequential multicollinearity (O’Brien, 2007). Multicollinearity was not considered problematic since the VIF values ranged from 1.38 to 6.11.

### Model Estimation and Evaluation

Model estimation was performed using the *lavaan* package (Rosseel, 2012) running in the *R* environment (R Development Core Team, 2011). The models were fitted with the maximum likelihood estimator, the most widely used in SEM. This estimator is unaffected by data transformation and has asymptotic properties, hence the minimum variance, unbiasedness, efficiency and consistency (Myung, 2003). The maximum likelihood estimator assumes multivariate normality but is appropriated and robust even if non-normality exists (Yuan and Bentler, 2005) and still produces centered estimates of the parameters (Finney and DiStefano, 2006) unless sk and ku are too severe (Kline, 2011), which was not the case for our data.

The model fit was assessed using the goodness-of-fit chi-square (χ^2^) statistic at a significance level α = 0.05. This model test statistic tests the null hypothesis that the population covariance matrix does not differ significantly from the model-implied covariance matrix. A failure to reject the null hypothesis means that the model is consistent with the sample data matrix, thus supporting the model assumptions. χ^2^ is the most commonly used statistic for the model fit in SEM to appraise the overall model fit (Hu and Bentler, 1999) and it works particularly well for sample sizes between 75 to 200 cases (Kenny, 2015). It tends to be significant for larger sample sizes due to its sensitivity to sample size (Schumacker and Lomax, 2010). Additionally, the correlation residuals of the model fit were established to confirm the explanatory power of the model for the specific associations observed. As a rule of thumb, a correlation residual with an absolute value greater than 0.10 is a sign of a poor explanation of the corresponding sample correlation (Schumacker and Lomax, 2010). In addition to χ^2^, several other approximate fit indexes were considered to provide further insights about the model fit such as the Steiger-Lind Root Mean Square Error of Approximation (RMSEA; Steiger, 1990), the Jöreskog-Sörbom Goodness of Fit Index (GFI; Jöreskog and Sörbom, 1982), the Bentler Comparative Fit Index (CFI, Bentler, 1990), the Tucker-Lewis Index (TLI; Tucker and Lewis, 1973), the Standardized Root Mean Square Residual (SRMR) and the ratio χ^2^/df. Taken together, these are among the most common approximate fix indexes that exist in the SEM literature (McDonald and Ho, 2002).

A multigroup analysis was also performed to determine how the considered disturbance factors specifically affect each riparian succession phase. This analysis was preceded by an evaluation of model measurement invariance, to judge the validity of the proposed comparisons, along with the computation of CFI variation (ΔCFI) to confirm the evaluation. During the multigroup analysis, the proposed models were individually fitted to each succession phase because the sample size of each group was considerably lower than the recommended sample size for these analyses. Consequently, a 95% confidence interval for the mean value of the group-specific model parameters was built in these cases based on larger sample sizes generated by the bootstrapping resampling method with 1000 bootstrap draws. All of the relationships between variables were analyzed using standardized coefficients for a better understanding of the direct effects between the variables (Grace and Bollen, 2005).

## Results

### Patch Location

**FIGURE 3:**
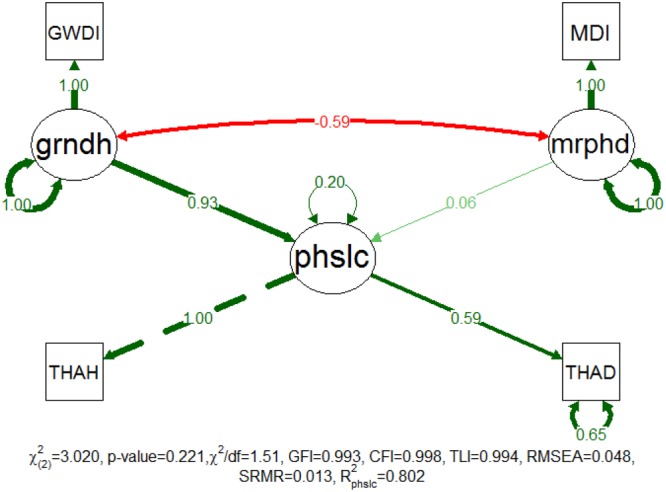
Completely standardized solution of the fitted model for patch location. Standardized path coefficients are shown in red or green accordingly to the sign of the relationship. Color intensity and arrow thickness are proportional to relationship magnitude. Continuous and discontinuous arrows stand for free and fixed-unit path coefficients. GWDI is an abbreviation for groundwater depth index, MDI for morphodynamic disturbance index, grndh for groundwater hydrology, mrphd for morphodynamic disturbance, phslc for phase location, THAH for height above thalweg, and THAD for distance to thalweg.

**Figure 3** shows the proposed model construct for patch location, which was successfully fitted with a non-significant χ^2^ test (*p*-value = 0.221). Accordingly, the population covariance matrix was not significantly different from the covariance matrix estimated by the model at a confidence level of 95%. The additional approximate fit indexes also supported this outcome and provided a perception of the goodness of the adjustment of the proposed model for the sampled data. The CFI, TLI, GFI and RMSEA indicated a very good adjustment, but the SRMR and χ^2^/df indicated that this result was a good adjustment (see Schumacker and Lomax, 2010; Kline, 2011; Marôco, 2014 for summaries on the fit classification indexes). Furthermore, all of the correlation residuals were less than 0.10, which suggested that the model could properly explain the corresponding sample correlations.

In this model, mrphd is manifested on MDi and grndh on GWDi. The latent variable of patch location is manifested on patch distance (THAD) and height (THAH) above thalweg, which indicates the patch positioning along the river stretches. The fitted model explains approximately 80% of the patch location variability, which implies that these variables contribute a considerable amount of information regarding patch location. The results show that the location of the riparian patches is primarily driven by grndh (standardized path coefficient = 0.930), whereas the mrphd seems of very little importance (standardized path coefficient = 0.061) to patch location. Regardless, grndh and mrphd are moderately correlated (-0.592). Consequently, riparian patches located at larger horizontal and vertical distances from the river thalweg appear to be subject to greater grndh disturbances. Furthermore, these results show that changes in grndh may lead to shifts in the location of succession phases, the eventual loss of the more vulnerable ones and the following extinction of its characteristic species.

The results for measurement invariance show that the patch location model indicates a weak invariance between study sites (H0: the model is invariant between study sites, not rejected for weak invariance with a *p*-value = 0.335). This finding means that the model construct is similar at all of the study sites and that for a given indicator, the factor loadings are significantly the same between the study sites. The ΔCFI (0.001) for the loadings is less than 0.01, indicating once more that invariance should not be rejected (Cheung and Rensvold, 2002). The model shows strong measurement invariance for the succession phases (the *p*-values for the weak and strong invariance tests are 0.443 and 0.167, respectively). The ΔCFI for the loadings and intercepts is correspondingly, 0.001 and 0.004, again smaller than the proposed threshold of 0.01 and therefore support the results of the invariance tests. A weak invariance allows the comparison of the relationships between the latent variables across groups while a strong invariance allows for the inter-group comparison of latent variable means and covariances (Chen, 2008).

**FIGURE 4:**
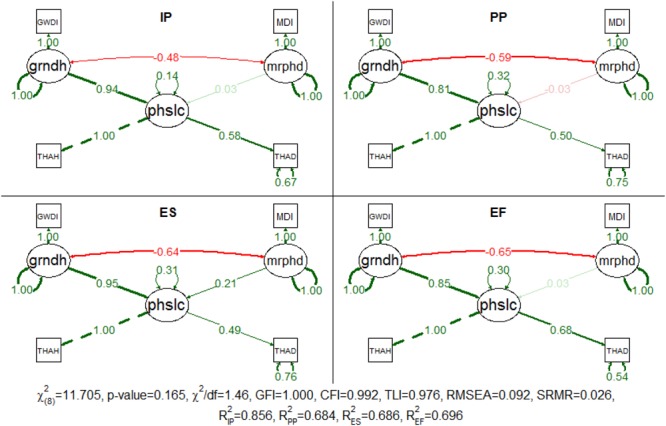
Completely standardized solution for the fitted model for patch location in each succession phase (IP, initial phase; PP, pioneer phase; ES, early succession phase; EF, established forest phase). Standardized path coefficients are shown in red or green accordingly to the sign of the relationship. Color intensity and arrow thickness are proportional to relationship magnitude. Continuous and discontinuous arrows stand for free and fixed-unit path coefficients. GWDI, groundwater depth index; MDI, morphodynamic disturbance index; grndh, groundwater hydrology; mrphd, morphodynamic disturbance; phslc, phase location; THAH, height above thalweg; THAD, distance to thalweg.

The proposed model was successfully fitted in the multigroup analysis (**Figure 4**). The χ^2^ test was not significant (*p*-value = 0.165) and the approximate fit indexes indicated the good adjustment of the model. The model explained more than 68% of the data variance, for group sample sizes of 74, 52, 72, and 22, respectively, for IP, PP, ES, and EF. As in the general location model, for every succession phase, both disturbances are still well correlated and the grndh is the main driver of patch location (the standardized path coefficients were greater than 0.81 for all succession phases). In contrast, the mrphd had a residual effect on the succession phases, except for ES (standardized path coefficient = 0.207), for which an increase in the mrphd determined greater distances to thalweg. This means that mrphd pushes away this succession phase to a more distant location, thus preventing vegetation encroachment. In detail, IP is characterized by recently disturbed patches where woody vegetation is starting to establish. The seedlings survive according to the recruitment box (Mahoney and Rood, 1998) and therefore, the link with the water table elevation is tight. If the recruitment survives, the patches evolve to PP after approximately 2 years. Individuals have now a settled root system but still vegetate inside the active channel, where the groundwater remains within reach almost all year round, accounting for the less pronounced link with the grndh. The ES patches live in a survival limbo between the grndh and mrphd. The indicator species of this succession phase (*Salix* spp.) are obligate phreatophytes well adapted to flow disturbances (González et al., 2010), whose existence is compelled by mrphd to the limit of the tolerated distance from the groundwater table. Consequently, these patches are considerably more likely to be negatively affected by groundwater level fluctuations and are therefore highly dependent on grndh. In turn, the typical facultative phreatophytes that characterize EF confer to this succession phase less dependency on this factor and a survival advantage over previous succession phases.

The 95% confidence intervals of the bootstrapped coefficients confirm the discrepancy in the effect between the grndh and mrphd on the location of the succession phase patches (**Table 2**). In fact, the magnitude of the effects of the two disturbances is completely distinct in the first three succession phases and always higher for grndh in all succession phases. Furthermore, one cannot rule out the possibility of that a mrphd would have no effect on the location of the succession phases, unlike grndh, which can be the only driving factor of the location of the succession phases as indicated by the proposed model. Succession phases have different heights and distances to thalweg, so it is possible to infer the location of the succession phases along the river stretch despite some degree of overlap. IP is the closest succession phase to the thalweg (the standardized values for mean distance and mean THAH is 3.220 and 3.406, respectively), followed by ES (4.669 and 4.731), PP (4.787 and 5.984) and EF in the outer parts of the river (5.499 and 6.824).

**Table 2:** 95% confidence intervals of the bootstrapped coefficients for the multigroup patch location model.

	IP	PP	ES	EF
Patchloc∼Groundhydro	0.848; 1.025	0.531; 1.364	0.615; 1.080	0.267; 1.149
Patchloc∼Morphodist	–0.130; 0.191	–0.406; 0.503	–0.005; 0.413	–0.379; 0.398
THAD	2.568; 4.242	3.831; 6.475	3.770; 5.903	5.437; 5.561
THAH	2.861; 4.216	5.393; 6.029	3.556; 6.253	6.743; 6.905

### Patch Shape Model

**FIGURE 5:**
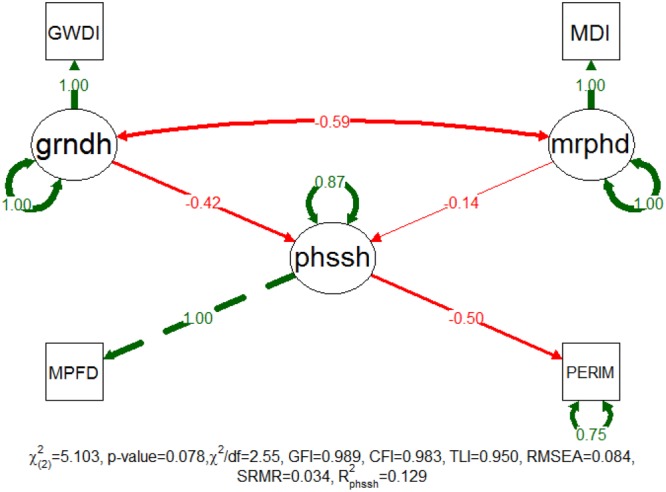
Completely standardized solution of the fitted model for patch shape. Standardized path coefficients are shown in red or green accordingly to the sign of the relationship. Color intensity and arrow thickness are proportional to relationship magnitude. Continuous and discontinuous arrows stand for free and fixed-unit path coefficients. GWDI, groundwater depth index; MDI, morphodynamic disturbance index; grndh, groundwater hydrology; mrphd, morphodynamic disturbance; phssh, phase shape; MPFD, mean patch fractal dimension; PERIM, patch perimeter.

The patch shape model was successfully fitted with a non-significant χ^2^ test (*p*-value = 0.078), and the additional approximate fit indexes indicated a good adjustment classification (**Figure 5**). GFI, CFI, TLI and SRMR indicated that this adjustment was very good while χ^2^/df and RMSEA classified it as acceptable. The latent variables of fluvial disturbance are manifested as GWDi and MDi while the latent variable patch shape is manifested as patch PERIMTR and the MPFD. Nevertheless, this model only explained approximately 13% of the patch shape variability and the disturbances accounted for have smaller effect on this feature (the sum of standardized patch coefficients is 0.562). The grndh continues to be the main driver of the two disturbance factors, despite that the effect of the mrphd (standardized path coefficient = -0.139) has now proportionally increased to approximately a third of the effect of the grndh (standardized path coefficient = -0.423).

The investigation of the measurement invariance revealed a weak and strong invariance between study sites (*p*-values of 0.268 and 0.844), respectively. Likewise, the ΔCFI for loadings (0.001) and intercepts (0.001) were smaller than the proposed threshold of 0.01, providing confirmation of both levels of measurement invariance. The model only shows weak measurement invariance (*p*-value = 0.383) for the succession phases supported by a ΔCFI for the loadings of 0.0004.

Notwithstanding, the patch shape model did not achieve a successful fit in the multigroup analysis. The χ^2^ test was significant [χ^2^_(8)_ = 20.791 and *p*-value = 0.008], meaning that the population covariance matrix was significantly different from the model-estimated covariance matrix. Also the approximate fit indexes indicated adjustment problems with contradictory classifications, specifically, an unacceptable fit by the TLI (0.757) and RMSEA (0.171), an acceptable fit by χ^2^/df (2.60), CFI (0.919) and SRMR (0.056), and a perfect fit by GFI (1.000). In addition, the explanatory power of the model was very low for all of the succession phases (0.066 for IP, 0.048 for PP, 0.034 for ES and 0.225 for EF). Taken together, these results demonstrated that the proposed model did not adequately explain the patch shape dynamics of the riparian succession phases in the Mediterranean rivers.

## Discussion

The current study focused on understanding the drivers of landscape features of Mediterranean riparian vegetation on a local scale, specifically, the location and shape of the patches. The approach adopted relied on testing the existing theories about the local drivers of riparian vegetation and its succession phases. Accordingly, the model-implied covariance matrices of the hypothetical model constructs were tested with the population covariance matrices built from the vegetation data collected at four different Mediterranean case studies. Both models were successfully fitted, confirming that the structural equation models were consistent with the sample data and were therefore able to correctly characterize the riparian patch dynamics of the considered Mediterranean rivers. Nevertheless, the patch location and shape models had very dissimilar capabilities in explaining the data variability, indicating that it was possible that some patch features were more greatly dependent on the disturbances considered than other features. The patch location model had a very good fit to the data and a high coefficient of determination when compared to the average explanatory power in ecological research (Low-Décarie et al., 2014). Moreover, the model showed that the patch location was almost exclusively driven by the grndh. In fact, the removal of the mrphd factor from the model did not result in a significant difference between the models (H_0_: model and sub-model are not different, *p*-value = 0.557). The primacy of this driver was found for the patch location of each succession phase as well. Only the ES phase was noticeably negatively affected by mrphd, which appeared to be a key element for preventing vegetation encroachment by this succession phase. We must admit that we were fairly surprised with the outcome regarding the effect of morphodynamics on the very early succession phases, such as IP and PP, which we expected to be higher. The abrasion effect of suspended load and bed load along with floating debris is undeniably an important morphodynamic process that was neglected in these analyses, which could explain the low impact of morphodynamics in very young succession phases. Likewise, shear stress, the proxy for mrphd, does not accurately represent the side erosion process, which is also an important effect, at least for meandering rivers (Scott et al., 1996). Nevertheless, we were persuaded to attribute this circumstance to the impact of extreme summer droughts that prevented the development of seedlings and younger succession phases in the river channel, in addition to a winter flow that inhibited the establishment and overtake by xerophyte species. Furthermore, this outcome is in consensus with the current knowledge that the physical habitat shaping in the inner river zones occupied by younger succession phases is controlled by lower and more frequent floods while older succession phases are bridled by higher and less frequent floods (Wolman and Miller, 1960), such as the maximum annual discharges used to characterize MDi. Accordingly, it appears that in our typically Mediterranean study sites, the location of the succession phase patches are more a result of zonation driven by water scarcity than ecological succession driven by different fluvial disturbances. This may probably be a common characteristic of Mediterranean-climate regions, where seasonal water scarcity is common and riparian ecosystems have similar adaptations and strategies to cope with the particular stresses of these analogous med-rivers (Bonada and Resh, 2013). Given this generalization, this circumstance seems to be one more particularity of Mediterranean riparian woodlands, because in temperate and tropical river systems the landform processes are considered to be the main driving factor of riparian vegetation (see Stromberg et al., 1996, 2007; Scott et al., 1999; Shafroth et al., 2000; Steiger et al., 2005). Although, other factors such as river geomorphology may prompt this divergence. Actually, all of the case studies considered were in a valley confined to some extent, with bed material ranging from bedrock to cobles, which may preclude channel movement and sediment transport, resulting in a much more stable river channel with low sinuosity in which broad processes of erosion/sedimentation occur with much more difficulty (Dingman, 2009). Soil composition has also been pointed as determinant in riparian landscape characterization (e.g., Molina et al., 2004) but in our case no linear relation was found between bed substrate and succession phases or their location. Furthermore, we considered substrate to be a result of fluvial disturbance rather than a component of it. For these reasons, we did not include bed substrate as a latent variable in our models. Nevertheless, the outcome of this analysis may still serve conservation and management purposes. In addition to the creation of the necessary mrphd to prevent vegetation encroachment and to preserve the naturalness of the riparian landscape, particularly in the younger succession phases, such results point to the necessity of maintaining a minimum river discharge capable of sustaining water table levels. Such sustenance can in fact prevent the rearrangement of the riparian succession phases following the increased water stress imposed by river regulation. Furthermore, it may also help in preventing the invasion by exotic species, as those appear to be able to adjust faster to new disturbance regimes than native species (Planty-Tabacchi et al., 1996). But in the end, the findings of this work are consistent with observations in previous studies in Mediterranean climate rivers, where changes in water table levels determined the rearrangement of the riparian communities according to the hydrologic thresholds of the species (e.g., Stromberg et al., 1996, 2007; Scott et al., 1999; Shafroth et al., 2000). Notwithstanding, the novelty of this study was the quantification of the importance of each fluvial disturbance in the landscape features of riparian vegetation.

As for the shape of the patches, grndh is still its major driver, even though mrphd were shown to have a much more important role, where greater distances to the water table elevation account for larger and less complex patches. However, the disturbances reported cannot be considered to be substantial drivers of this feature, as indicated by the very low explanatory power of this model. Although common in ecological models (Møller and Jennions, 2002), this indicates that the majority of the patch shape variation comes from the residual variance of the model, suggesting that other factors are more influential in riparian patch shaping. Notwithstanding, there is still no consensus about the main drivers of this feature. Other factors such as geomorphology, human impact, valley width or stream sinuosity have been implicated in previous studies (e.g., Fernandes et al., 2011; Aguiar et al., 2016; Zhou et al., 2016), although not using a confirmatory factor analysis, but which can certainly be effective driving forces of patch complexity.

## Conclusion

Fluvial disturbances were demonstrated to have different effects on the location and shape of riparian vegetation patches. The main driver of riparian patch location was grndh and indicated the predominant zonation of riparian succession phases over natural ecological succession. Nevertheless, mrphd are still responsible for preventing vegetation encroachment. However, patch shape seemed not to be primarily driven by fluvial disturbances but, within the limited explained variability of the proposed model, both grndh and mrphd had a substantial impact. These outcomes emphasize the likely necessity for specific procedures during flow regime management to account for the particularities of the drivers of fluvial disturbances of riparian vegetation in Mediterranean rivers.

## Author Contributions

RR established the experimental design, conducted the experiment, analyzed data and wrote the manuscript. AP supervised the hydraulic modeling and reviewed the manuscript. GE supervised the ecological interpretation of the results and reviewed the manuscript. TF was the main supervisor and reviewed the manuscript.

## Conflict of Interest Statement

The authors declare that the research was conducted in the absence of any commercial or financial relationships that could be construed as a potential conflict of interest.

## Abbreviations

CFI: comparative fit index
EF: established forest phase
ES: early successional woodland phase
GFI: goodness of fit index
grndh: groundwater hydrology
GWDi: groundwater depth index
IP: initial phase
ku: kurtosis
MDi: morphodynamic disturbance index
MPFD: mean patch fractal dimension
mrphd: morphodynamic disturbance
PERIMTR: perimeter
phslc: patches location
phssh: patches shape
PP: pioneer phase
RMSEA: root mean square error of approximation
SEM: structural equation modeling
sk: skewness
SRMR: standardized root mean square residual
THAD: distance to thalweg
THAH: height to thalweg
TLI: Tucker-Lewis Index
VIF: variance inflation factor
